# Childhood necrotising pneumonia, empyema and complicated parapneumonic effusion secondary to community acquired pneumonia*: report of 158 cases from a tertiary hospital in Egypt*

**DOI:** 10.1186/s12931-025-03291-w

**Published:** 2025-07-02

**Authors:** Salma Abdelhady, Amira A. Moharram, Zainab Fawzy, Eman Fouda

**Affiliations:** 1 Department of Paediatrics, Faculty of Medicine, Ain Shams University, Cairo, Egypt; 2 Paediatric Respiratory department, Children’s Hospital, Ain Shams University Hospitals, Cairo, Egypt; 3 Department of Clinical Pathology, Faculty of Medicine, Ain Shams University, Cairo, Egypt

**Keywords:** Community acquired pneumonia, Complicated pneumonia, Parapneumonic effusion, Pleural effusion, Empyema, Necrotizing pneumonia, Necrotising pneumonia, Pleural fibrinolytics, COVID-19, Children

## Abstract

**Background:**

Incidence of childhood complicated community acquired pneumonia (cCAP) is increasing worldwide. Necrotising pneumonia (NP), empyema and complicated parapneumonic effusion (CPPE) are the most common local complications.

**Methods:**

This retrospective observational study describes clinical characteristics, aetiology and management of children hospitalized with cCAP in one of the largest tertiary centers in Egypt, over 5 years (December 2017 till September 2022).

**Results:**

A total of 158 cases were identified. Seasonal variation was observed, as more cases were hospitalized during Winter and Spring. NP, empyema and CPPE, were diagnosed in 85 (54%), 52 (33%) and 21 (13%) children, respectively. 54 (64%) of children presented with NP had associated empyema or CPPE. The yield of pleural fluid, sputum and blood cultures were 23%, 18% and 17%, respectively. Community acquired *MRSA* was the predominant causative organism, followed by *S pneumoniae*. 87% of the patients had pleural interventions. 29 (18%) children received fibrinolytics. Three children presented with CAP and highly septated effusion, developed NP and persistent air leaks following fibrinolytic administration. Patients had prolonged hospitalization (median 17 days). 15 (10%) children had surgery. Children presented with NP had more morbidities and longer length of hospital stay, compared to children presented with CPPE and empyema. ICU admission, mechanical ventilation, severe anemia requiring blood transfusion, broncho-pleural fistula and surgical interventions were significantly higher in NP cohort. We report 5 mortalities, 4 of them below 1 year of age.

**Conclusions:**

This study describes the largest cohort of children hospitalized with cCAP from Egypt till this date. Management of cCAP remains challenging worldwide and the current guidelines requires updating. Improvement of microbial detection and reporting is needed to promote antimicrobial stewardship.

**Supplementary Information:**

The online version contains supplementary material available at 10.1186/s12931-025-03291-w.

## Introduction

Community-acquired pneumonia (CAP) is the leading cause of morbidity and mortality worldwide in children aged between 1 month and 5 years [1]. Globally, CAP affects approximately 120–150 million children annually, with the highest burden observed in low- and middle- income countries [2–4]. In children under the age of five, CAP accounted for an estimated 693,000 deaths in 2019 and remained a major cause of mortality in 2021, with approximately 502,000 deaths globally [2–4]. In Egypt, CAP is responsible for about 10% of childhood mortality under the age of five [5]. Despite the high burden of CAP in Egypt, data on the incidence of cCAP remain scarce.

Around 3–7% of pediatric CAP becomes complicated [6–8], and the high frequency of pneumonia in children worldwide makes its complications a significant problem. Complicated community acquired pneumonia (cCAP) in children frequently presents with severe illness and significantly prolonged hospital stay compared with CAP [6]. Although most children fully recover, however, childhood pneumonia has been associated with long-term pulmonary complications, including a higher risk of bronchiectasis, restrictive lung disease, and the development of asthma in adulthood [9].

Complicated Community-acquired pneumonia consists of one or more of pleural effusion, empyema, lung abscess, and necrotising pneumonia; the local CAP complications. Pleural effusion is the most common complication of CAP and can be divided into three stages: exudative (simple parapneumonic effusion), fibrinopurulent (complicated parapneumonic effusion), and the organizing phase with fibrosis and peel formation [6]. Complicated parapneumonic effusion (CPPE) is characterized by the formation of septations and loculations within the pleural cavity. Empyema is defined as pus, on gross appearance, during thoracentesis [6]. Necrotising pneumonia (NP) is characterized by liquefaction of the lung parenchyma and formation of multiple air-filled cavities (pneumatoceles) [8, 10, 11]. Pneumatoceles are thin-walled, air-filled intraparenchymal cavities that develop secondary to localized bronchiolar and alveolar necrosis, which allow one-way passage of air into the interstitial space. These necrotic cavities can coalesce and form large cysts that can rupture into the pleural space causing pneumothorax or bronchopleural fistula (persistent air leaks) [8, 10, 11]. Radiological imaging is necessary for the diagnosis of NP [8, 12]. *S pneumoniae* is the most common cause of necrotising pneumonia in children [6, 8]. Severe forms of necrotising pneumonia have been associated with *S aureus* strains expressing Panton- Valentine leucocidin, a pore-forming exotoxin that lyses immune cells, potentially releasing tissue-damaging proteases [13]. Additionally, bacterial pore-forming toxins, such as *Streptococcus pyogenes*-derived streptolysin O and *Staphylococcus aureus*-derived α-hemolysin, promote neutrophil–platelet aggregation, which may drive microvascular thrombosis and alveolar-capillary destruction in necrotising pneumonia [6, 14, 15].

Systemic complications associated with CAP include sepsis, septic shock, multiorgan failure, acute respiratory distress syndrome, disseminated intravascular coagulation, and death [6].

*Streptococcus pneumoniae (S pneumoniae)*, *Streptococcus pyogens (S pyogens)*, *Staphylococcus aureus (S aureus)* are the most common pathogens associated with cCAP in children. Introduction of pneumococcal conjugate vaccine 13 (PCV13) has led to reduction of invasive pneumococcal disease (IPD) burden in children, with simultaneous rise of cCAP caused by *S pyogens* and *S aureus* [16–20], this may be influenced by advancements in diagnostic methodologies, warranting careful interpretation of epidemiological trends. In Egypt, pneumococcal conjugate vaccine (PCV) is not included in the childhood mandatory vaccinations, and it is not widely used. This hypothetically poses a greater risk of CAP and its complications in Egyptian children.

Till this date, management of cCAP remains problematic and treatment strategies are often chosen on case-by-case basis. Prolonged systemic antimicrobial therapy and local interventions remain the standard of care. Choice of antibiotics is guided by local microbiological knowledge and culture results. However, data from low and middle-income countries about most common pathogens and antimicrobial resistance with focus on pediatric cCAP is sparse [21]. This contributes to a greater challenge for cCAP management. Therefore, our study aimed at evaluating the incidence, clinical presentation, causative organism, treatment strategies and outcomes of cCAP in children treated at one of the largest tertiary hospitals in Cairo, Egypt.

## Materials and methods

### Study subjects and data collection

The study was carried out at the pediatric pulmonology unit, Children’s hospital, Ain Shams University, Cairo, Egypt. Records of children hospitalized with cCAP (CPPE, empyema and NP) between December 2017 and September 2022 were reviewed. Children admitted with pleural effusion related to pulmonary or extra pulmonary diseases other than CAP were excluded. Patients hospitalized with lung abscess were also excluded from this study. The screening of hospital records was conducted manually by author SA and independently reviewed by author EF to ensure data accuracy and consistency. The study was approved by the ethical committee of Ain Shams University hospitals (ethical approval number FMASU R252/2023).

A retrospective hospital records review was performed using a standardized data collection form. For each patient, demographic data, clinical presentation, admission laboratory results including full blood count, CRP, microbiological culture & sensitivity, and COVID-19 RT-PCR results, were recorded. Treatment strategies (antibiotic treatment, chest drainage and pleural fibrinolytic therapy), duration of hospital admission and treatment outcomes were also recorded.

As per our hospital protocol, blood culture and senstivity was routinely performed for all the patients. Pleural culture and senstivity was done for all the patients who had chest drainage or thoracocentesis. Sputum culture and senstivity was sent if the patient was able to give an adequate sputum sample, and for patients with severe clinical presentation requiring PICU admission. Sputum samples were collected following standardized clinical protocols. Patients were instructed to produce deep cough sputum, avoiding saliva and postnasal secretions, and specimens were collected in sterile, screw-capped containers. For intubated patients, endotracheal aspirates were obtained using sterile sputum traps and transferred aseptically to sterile containers.

### Statistical analysis

The statistical analysis of the data was performed using SPSS version 20.0 software. Categorical variables were analyzed using the Chi-square test and were expressed as frequency and percentage. The quantitative data were presented as median and interquartile range (IQR). Mann– Whitney U test was used to examine the differences in quantitative parameters between the groups in this study.

## Results

**Fig. 1:**
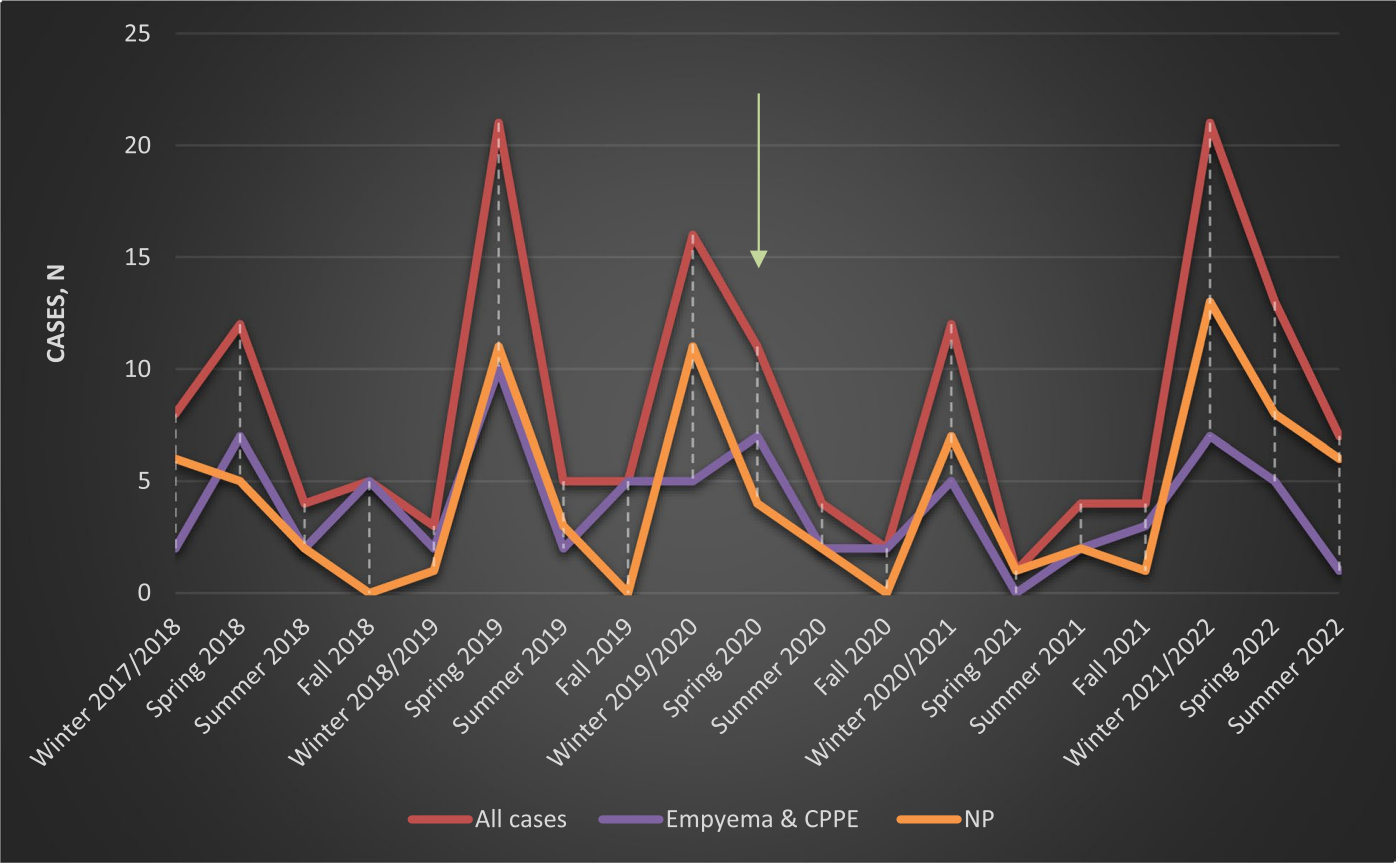
Number of children presented with CPPE, Empyema and NP differed through different seasons. There is observed peaks during Winter and Spring, with exception of Spring 2020 and Spring 2021 following the 2 lockdown periods of the 1 st and 2nd COVID-19 waves. **Note the Arrow** marks the start of the 1 st COVID wave, Spring 2020. Also, note the increase in NP admissions over the years

One hundred and fifty-eight children with cCAP were hospitalized between December 2017 and September 2022. Complicated parapneumonic effusion (CPPE), empyema and NP were diagnosed in 21 (13%), 52 (33%) and 85 (54%) children, respectively. All patients with CPPE and empyema had evidence of underlying pneumonia on imaging. No cases of isolated pleural infection were identified. Majority of the children presented with NP had associated CPPE or Empyema (64%, 54 out of 85). cCAP admissions peaked during Winter and Spring seasons. Seasonal variation and impact of COVID-19 on cCAP admissions are shown in Fig. 1.

Most patients had no significant prior medical history. Sixteen (10%) patients had failure to thrive, 7 (4%) had BMI Z-score > + 2 Standard deviation, and 8 (5%) patients had underlying neurological or neurometabolic disease. Three children (2%) were diagnosed with primary immune deficiency after the diagnosis of complicated pneumonia, and 1 child (0.6%) had chronic kidney disease.

Systemic complications were observed in 12 patients (8%). Seven patients had septic shock, 3 had carditis and mild pericardial effusion, 1 patient had deep venous thrombosis and 1 patient had osteomyelitis and syndrome of inappropriate ADH secretion.

The median age at presentation was 3.5 years (range 0.2–15 yrs.). Study cohort was classified into 2 groups: group 1 (children who had CAP and Empyema or CPPE) and group 2 (children who had NP)**.** The diagnosis of NP was done based on the chest imaging. Patients were included in group 2 if they had NP on admission or developed NP during hospitalization. Majority of the patients had fever before hospital admission (98%, 156 out of 158). Cough, dyspnea and abdominal pain were the most common presenting symptoms. Clinical and demographic data are illustrated in Table 1.

**Table 1:** Demographic and clinical characteristics of the study cohort

	**Group 1 (*n* = 73) (Empyema/CCPE)**	**Group 2 (*n* = 85) (NP)**	**All cases (*n* = 158)**	***P* value**^¶^**(Significance)**
**Median Age in years (Range)**	4 (0.2–13)	2.8 (0.2–15)	3.5 (0.2–15)	0.105 (NS)
**Gender (%)**				
Female	53 (73%)	41 (48%)	94 (60%)	0.002 (HS)
Male	20 (27%)	44 (52%)	64 (40%)	
	**Median (IQR)**	**Median (IQR)**	**Median (IQR)**	
**Hospital stay in days**	14 (12–21)	21 (14–30)	17 (14–24)	0.001 (HS)
**Duration of drainage in days**	7 (6–10)	13 (7–24)	10 (6–16)	0.002 (HS)
**Duration of illness before hospital admission**	13 (7–17.5)	12.5 (6–15)	13 (7–15)	0.438 (NS)
	**No. (%)**	**No. (%)**	**No. (%)**	
**Symptoms**				
Cough	53 (73%)	47(55%)	100 (63%)	0.030 (S)
Dyspnea	55 (75%)	67(79%)	122 (77%)	0.507 (NS)
Abdominal pain	18 (25%)	10 (12%)	28 (18%)	0.037 (S)
Vomiting	6 (8%)	8 (9%)	14 (9%)	0.775 (NS)
Diarrhea	1 (1%)	6 (7%)	7 (4%)	0.080 (NS)
Constipation	3 (4%)	3 (4%)	6 (3%)	0.861 (NS)
Chest pain	4 (6%)	0 (0.0%)	4 (2%)	0.030 (S)
**Antibiotics before hospital admission**	61 (84%)	73 (86%)	134 (85%)	0.685 (NS)
**PICU admission**	13 (18%)	33 (39%)	46 (29%)	0.004 (HS)
**Ventilation**	3 (4%)	12 (14%)	15 (10%)	0.032 (S)
**Blood transfusion**	16 (22%)	31 (37%)	47 (30%)	0.046 (S)
**Chest drainage**				
No drainage	10 (14%)	10 (12%)	20 (13%)	
Pigtail	51 (70%)	10 (12%)	61(39%)	
Chest tube (Thoracostomy tube)	9 (12%)	56 (66%)	65 (41%)	
Therapeutic Thoracocentesis	3 (4%)	2 (2%)	5 (3%)	
Pigtail then chest tube	0 (0.0%)	7 (8%)	7 (4%)	
**Pleural Fibrinolytic therapy**	23 (32%)	6 (7%)	29 (18%)	0.00 (HS)
**Bronchopleural fistula and PALS**	0 (0.0%)	30 (35%)	30 (19%)	0.00 (HS)
**Surgery**	3 (4%)	12 (14%)	15 (10%)	0.032 (S)

Seven patients presented with severe pneumonia and associated effusion on admission required change in drainage from pigtail to surgical chest drain, as they developed pneumothorax and PALS during admission

64% of children presented with NP had associated CPPE or Empyema (54 out of 85)

^¶^*P*-value > 0.05: Non-significant (NS); *P*-value < 0.05: Significant (S); *P*-value < 0.01: highly significant (HS)

Children presented with NP or developed NP during admission (Group 2) had a more complicated hospital course compared to children presented with CAP and empyema or CPPE (Group 1). Children in NP cohort had a significantly longer hospital stay and duration of chest drainage. Additionally, PICU admissions, need for ventilation, blood transfusion and surgical interventions were significantly higher in NP group. More than half of the children in NP cohort had pyo-pneumothorax (55%, 47 out of 85). Five percent of children with NP presented with pneumothorax (4 out of 85). Broncho-pleural fistula (BPF) and persistent air leaks (PALS) were observed only in NP cohort (35%, 30 out of 85).

### Laboratory tests

There were no statistically significant differences in the laboratory tests between patients presented with CPPE/empyema and children presented with NP, except from the pleural fluid cell count and albumin. Admission laboratory results are shown in (Table 2). Ninety eight percent of study cohort had thrombocytosis during their course of illness (observed on admission or during the period of hospitalization).

**Table 2:** Laboratory data on admission represented as median (IQR)

	**Empyema/CCPE (*n* = 73)**	**NP (*n* = 85)**	**All cases (*n* = 158)**	*P*-value* (Significance)
**TLC (× 10**^**9**^**/L)**	17.1 (11.5–28)	19.8 (14.8–29)	19.5 (12.7–28.2)	0.404 (NS)
**Neutrophils (× 10**^**9**^**/L)**	10.2 (7–19.7)	12 (6.9–19.7)	11.1 (6.9–19.7)	0.640 (NS)
**Lymphocytes (× 10**^**9**^**/L)**	3.8 (2.5–6.5)	4.4 (2.8–7.7)	4.1 (2.6–7.1)	0.161 (NS)
**Hemoglobin (g/dl)**	9.7 (8.5–11)	9.9 (8.75–11.05)	9.8 (8.7–11)	0.499 (NS)
**CRP (mg/dl)**	187 (114–279.5)	160 (100–294)	180 (107–286.3)	0.787 (NS)
**Platelets (× 10**^**9**^**/L)**	472 (334–615)	523 (342–716)	495 (338–682)	0.288 (NS)
**S. Albumin (g/dl)**	2.9 (2.7–3.1)	3 (2.8–3.3)	2.9 (2.8–3.2)	0.131 (NS)
**Serum LDH (IU/L)**	410 (254–493)	377 (288–442)	378 (275.5–449.5)	0.934 (NS)
**Pleural Fluid Analysis**				
**Cell count (cells/ml)**	835 (360–4030)	335 (108–1150)	485 (175–1825)	0.038 (S)
**Albumin**	2.8 (2.4–3.4)	2.3 (2–2.7)	2.6 (2–3)	0.038 (S)
**LDH (IU/L)**	3781 (644–13500)	3444 (1323–10060)	3613 (994–12526)	0.965 (NS)
**Glucose (mg/dl)**	10.45 (1.7–23)	4.4 (1–12.3)	6.8 (1.35–20)	0.151 (NS)

^*^P-value > 0.05: Non-significant (NS); *P*-value < 0.05: Significant (S); *P*-value < 0.01: highly significant (HS)

### Microbiology

**Fig. 2:**
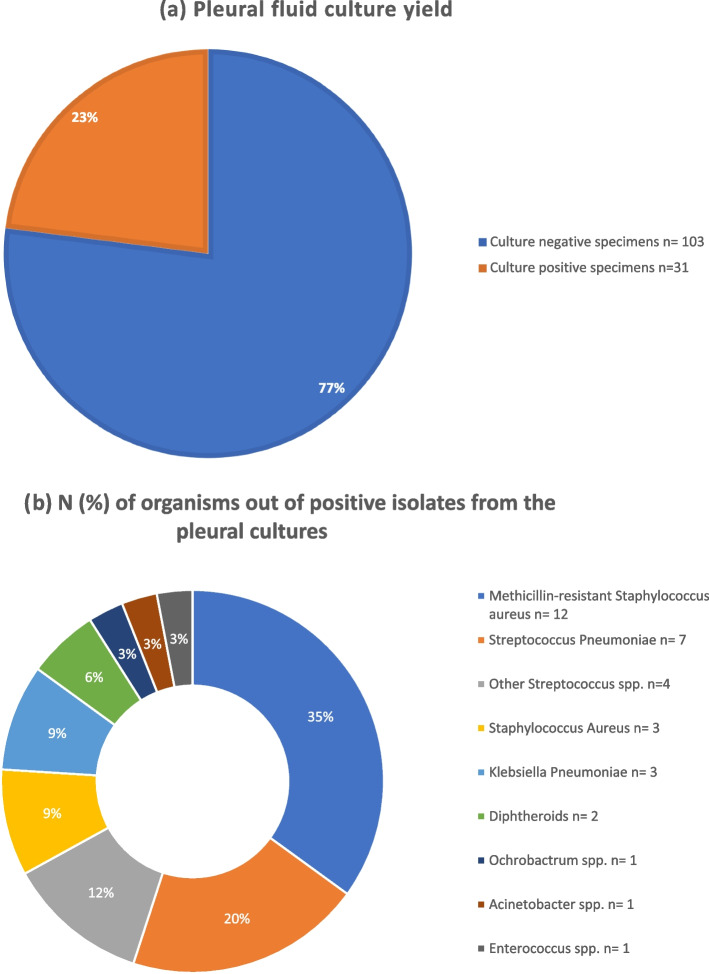
**a** Pleural fluid culture yield. **b** N (%) of organisms out of positive isolates from the pleural cultures

**Fig. 3:**
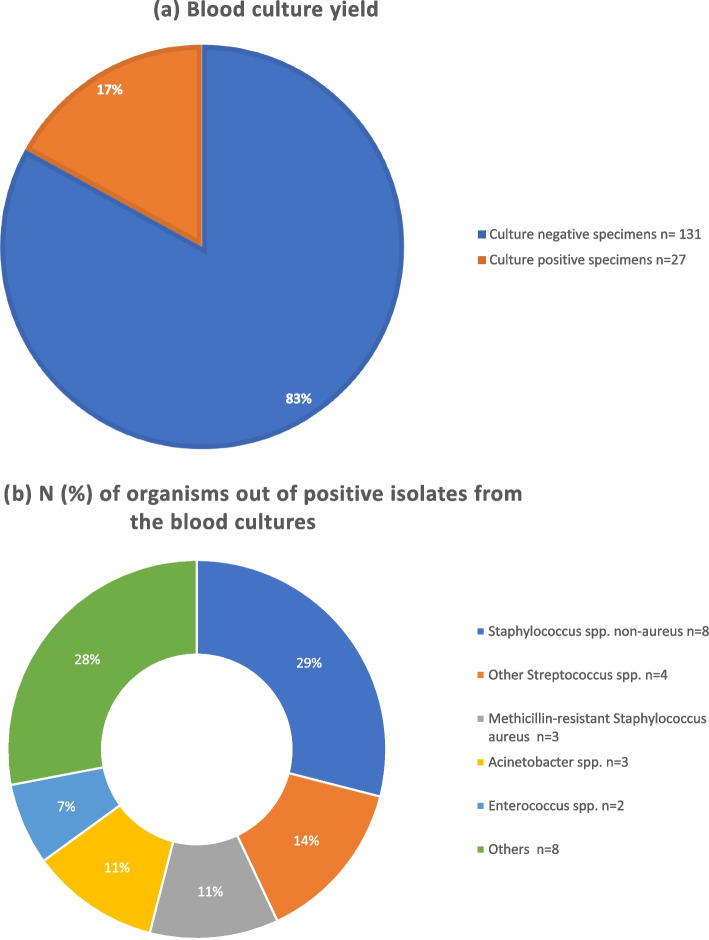
**A** Blood culture yield. **b** N (%) of organisms out of positive isolates from the blood cultures

**Fig. 4:**
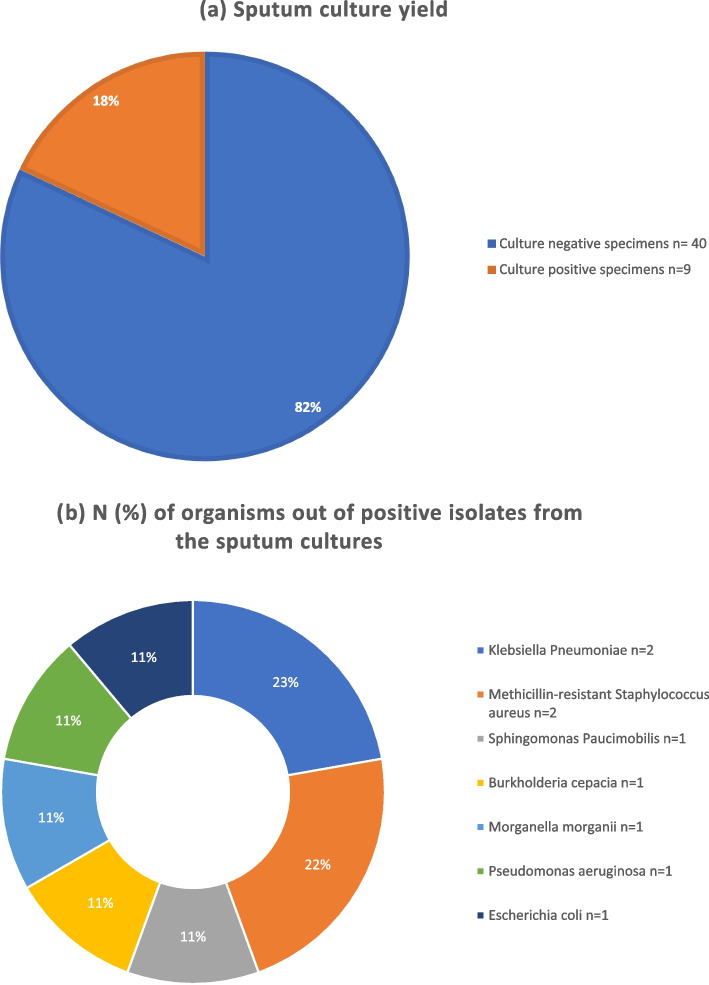
**a** Sputum culture yield. **b** N (%) of organisms out of positive isolates from the sputum cultures

The yield of pleural, sputum and blood cultures were 23%, 18% and 17% respectively. Microbial culture results are shown in Figs. 2, 3 and 4. Polymicrobial infections were noted in eleven patients (7%, 11 out of 158). We report one patient with negative bacterial cultures and positive gene Xpert for Mycobacterium tuberculosis on pleural fluid.

**Fig. 5:**
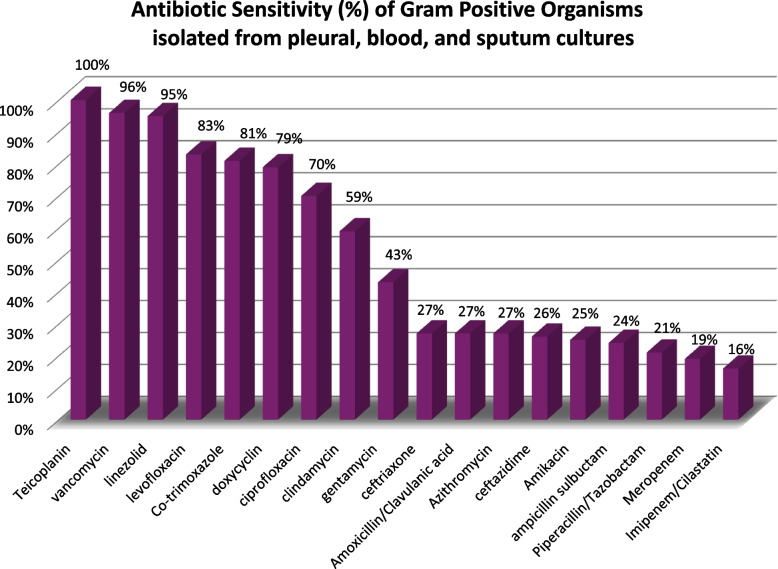
Antibiotic Sensitivity (%) of Gram Positive Organisms isolated from pleural, blood, and sputum cultures

**Fig. 6:**
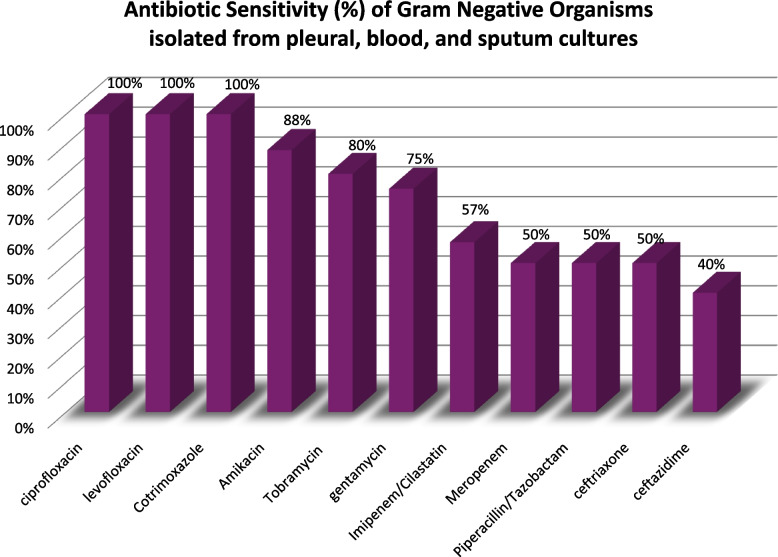
Antibiotic Sensitivity (%) of Gram Negative Organisms isolated from pleural, blood, and sputum cultures

Among our study cohort, the predominant Gram-positive organisms were *Methicillin-resistant Staphylococcus aureus (MRSA)* and *Streptococcus pneumoniae*, while *Klebsiella pneumoniae* was the leading Gram-negative organism. Isolated organisms were grouped to Gram positive and Gram negative organisms. Data on combined antimicrobial sensitivity from positive cultures is shown in Figs. 5 and 6.

Two patients had severe NP, BPF and persistent fever with raised inflammatory markers over a month after admission. All of their admission cultures were negative, and they had history of recurrent pneumonias and failure to thrive. Consultant decision was made to proceed for flexible bronchoscopy to investigate the cause of non-resolving pneumonia. Bronchoalveolar lavage culture showed growth of Klebsiella pneumonaie and S pyogens, respectively. Both patients showed clinical improvement on change of antibiotics based on the microbial sensitivity results.

Repeat cultures were obtained during admission if the patient showed clinical deterioration or had persistently elevated—or rising—inflammatory markers. In eight patients, the repeat cultures grew a different organism compared to the admission cultures. Specifically, repeat cultures identified multidrug-resistant (MDR) *Acinetobacter* in 50% (4 of 8), MDR *Klebsiella* in 25% (2 of 8), and MDR *Pseudomonas aeruginosa* in 25% (2 of 8). The results of these cultures were excluded from the microbial analysis. The authors engaged in several discussions regarding this subgroup. The microbiology team advised that the clinical significance of the repeat culture findings could not be definitively established, as long-stay patients may be asymptomatic colonizers. Furthermore, the relationship between these findings and length of stay (LOS) could not be determined due to multiple confounding factors, including varying antibiotic regimens, differing severity of pneumonia, organism virulence, and the presence of comorbidities.

### COVID-19 associated cCAP

**Fig. 7:**
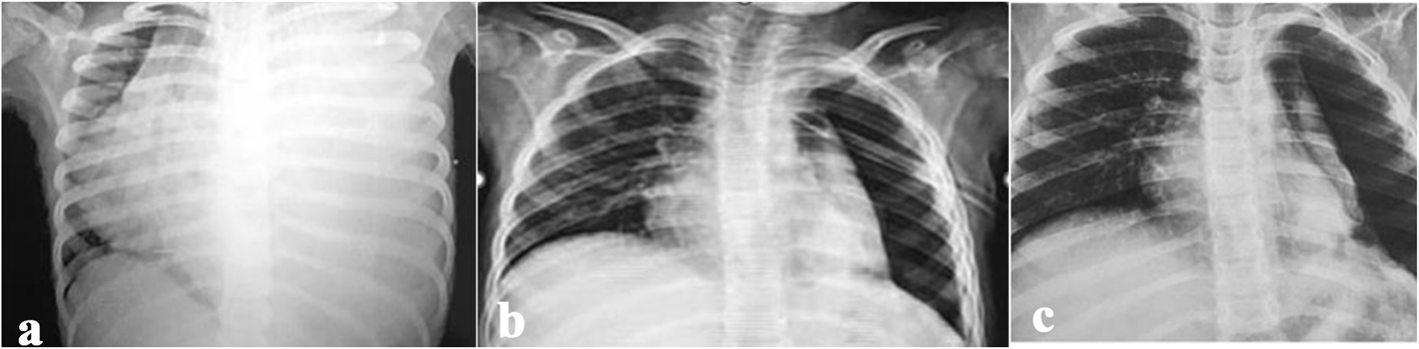
8-year-old female patient presented with grade III respiratory distress and **a**) chest X-ray showed massive left sided pleural effusion. Nasopharyngeal SARS-CoV-2 RT-PCR was positive. Chest drainage yielded 800 cc pus over 24 h. She also had persistent tachycardia, elevated troponins and echocardiography confirmed diagnosis of carditis and mild pericardial effusion. Two days later, she had worsening respiratory distress and CT chest showed evidence of air-filled cavities and pyo-pneumothorax confirming progression to necrotising pneumonia. She received systemic steroids, intravenous immunoglobulins, in addition to antibiotics. Pleural, blood and sputum cultures were negative. Despite 3 weeks of chest drainage and suction, she had persistent air leak and chest X-ray **b**) shows a trapped lung, 3 weeks after admission. Air leak resolved after 4 weeks and chest drain was removed, however, **c**) due to failure of lung expansion and persistent pneumothorax, she was referred for decortication

Six patients had COVID-19 associated cCAP (4%). The first child had COVID-19 associated NP & carditis (Fig. 7). The second child was known CKD patient, presented with left sided empyema. The third & fourth cases had minimal effusion associated with lobar pneumonia and bilateral bronchopneumonia respectively. All the previous cases had positive nasopharyngeal Covid-19 PCR on presentation and negative bacterial cultures. Fifth patient was a 3 month old infant who had a post COVID-19 empyema and multi-system inflammatory syndrome (MIS-C), with positive pleural culture showing growth of MRSA. All patients had radiological picture suggestive of complicated bacterial pneumonia and atypical for COVID-19, so, they were treated with antibiotics. Additional IVIG and steroids were given to patients with evidence of cardiac involvement. Chest drainage was inserted in 3 out of 5 cases with significant effusion. Outcome was favourable in 4 out of 5 cases with complete resolution. The 3 month old infant presented with empyema and MIS-C died due to sepsis and cardiac complications.

### Chest drainage

Most of the patients underwent procedural drainage (87%, 138 out of 158) (Table 1). Intervention radiology team performed ultrasound guided thoracocentesis or pig-tail insertion if the patient presented with moderate or severe effusion (> 2 cm on chest ultrasound). In case of patients presented with pneumothorax or pyo-pneumothorax, thoracic surgery team inserted a chest tube (thoracostomy tube) connected to an underwater seal system, under local anaesthetic. This explains the finding that most of the patients presented with empyema or CPPE had pigtails (70%, 51 out of 73) and most of patients who had NP had surgical chest drains (66%, 56 out of 85).

### Antibiotics and adjuvant therapies

Initial antibitoc protocol for cCAP on admission was 3rd generation cephalosporin (cefotaxime or ceftriaxone), plus, vancomycin or clindamycin. Most freqently found criteria for change in antibiotics on hospital records were persistent high-grade fever more than 72 h, rising inflammatory markers and clinical deterioration; after successful draiange of PE. Nevertheless, it was primalry the consultants’s decision. Eighty seven percent of the study cohort needed at least one change in antibiotics during admission. The most common second line antibiotics were meropenem, amikacin, linezolid and ciprofloxacin.

Seven patients had extensive NP, BPF and severe thrombocytosis (platelet count > 900 × 109/L), received antiplatelets (Aspirin 3 mg OD), as an adjuvant therapy to antibiotics and drainage. Low dose steroids (dexamethasone 0.3 mg BD), saline irrigation and suctioning were also used as adjuvant therapies in some patients, however, the data could not be correlated to clinical outcomes due to the retrospective nature of the study.

### Pleural fibrinolytics and their complications

Patients with moderate to severe septated effusion on LUS received fibrinolytic therapy (Alteplase), 4 mg diluted in 40 mL saline 0.9%, 2 doses 24 h apart. Twenty-nine patients (18%) among the study cohort received pleural fibrinolytics.

Three patients (10%) required blood transfusion following fibrinolytic administration due to significant hemoglobin drop. One patient (3%) had failed drainage and was referred for decortication.

Twenty-three (32%) of children in CPPE and empyema cohort (group 1) had septations on LUS and received fibrinolytic therapy. Subgroup analysis is shown in Table 3. Fibrinolytic therapy among patients with septated PE was found to accelerate fluid drainage and there was no statistically significant difference regarding duration of chest drainage and hospital stay between patients with septated PE who received fibrinolytic therapy and patients with non-septated PE who didn’t receive fibrinolytics.

**Table 3:** Subgroup analysis among Group 1 (fibrinolytics, Versus, no fibrinolytics)

**Group 1 (Empyema/CPPE)** ***n* = 73**	**Non septated effusion on LUS** **No fibrinolytics administered** ***n* = 44** Median (IQR)	**Septated effusion on LUS** **Received fibrinolytics** ***n* = 29** Median (IQR)	**Test value**	***P*-value**
Hospital stay (days)	14 (11–20)	14 (12—21)	−0.434	0.664*
Duration of drainage (days)	7 (5.5—12.5)	7 (6—10)	−0.262	0.793*

^*^Non -significant

**Fig. 8:**
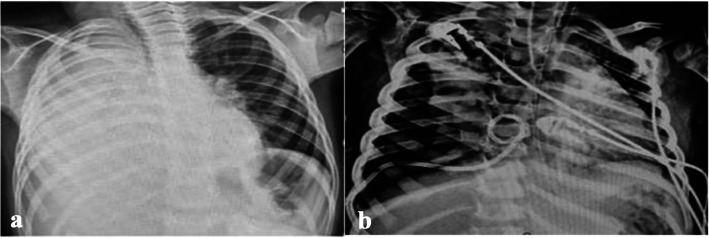
3-year-old male presented with **a**) severe pneumonia and associated effusion. Lung ultrasound showed highly turbid and septated effusion. **b **Following administration of intrapleural fibrinolytic, he developed air filled cavities, pneumothorax and worsening respiratory distress requiring escalation of respiratory support to high frequency oscillatory ventilation. Among the study cohort, we reported 3 patients with similar presentation of extensive consolidation and highly septated effusion, and all 3 developed air-filled cavities, BPF and PALS following fibrinolytic administration. This deterioration after fibrinolytics could be part of disease progression to necrotising pneumonia and coincided with fibrinolytics administration, however, we provide alternative hypothesis that administration of fibrinolytics destabilized the fibrinous inflammation which is part of inflammatory reaction in complicated pneumonia. We propose a slower rate of drainage than 10 ml/kg/hour in children, who present with massive effusion and significant underlying lung collapse

Fibrinolytics were generally avoided in patients presented with NP on admission. However, 6 out of 85 (7%) of children with NP (group 2) received fibrinolytics for management of associated septated effusion. Three children had NP and highly echogenic septated effusion on admission, despite this, consultant decision was to administer pleural fibrinolytics to drain the effusion after risk–benefit assessment. The other three patients initially presented with severe CAP complicated with empyema, and after administration of intrapleural alteplase they developed necrotizing pneumonia, pneumothorax, and bronchopleural fistula (Fig. 8). Notably their initial presentation showed severe consolidation with highly septated moderate to severe effusion. After intrapleural administration of alteplase and drainage of the effusion, they developed extensive parenchymal air-filled cavities that quickly progressed to pneumothorax, PALS and BFP. There is no conclusive evidence that this deterioration is secondary to pleural fibrinolytics, as it could be part of disease progression that coincided with alteplase administration.

### Outcome of hospitalisation

Fifteen patients (10%) were referred for surgery among the study cohort. Ten patients had decortication, 4 patients had decortication and lobectomy and 1 patient had video assisted thoracoscopic surgery (VATS). Referral to surgery was made after resolution of fever and normalization of blood inflammatory markers. Most of the children who needed surgery were in NP cohort.

We report 5 mortalities among the study cohort. 2-months-old infant who had concomitant RSV and MRSA infection, a 3-month-old infant who had a Post COVID-19 empyema and MIS-C, with pleural culture showing growth of MRSA. 7-month-old infant diagnosed with PID and 15-year-old boy with history of global developmental delay with pleural culture showing growth of Non-hemolytic Streptococcus. A 5-year-old girl with extensive NP and PALS, died 3 days after decortication and lobectomy.

**Fig. 9:**
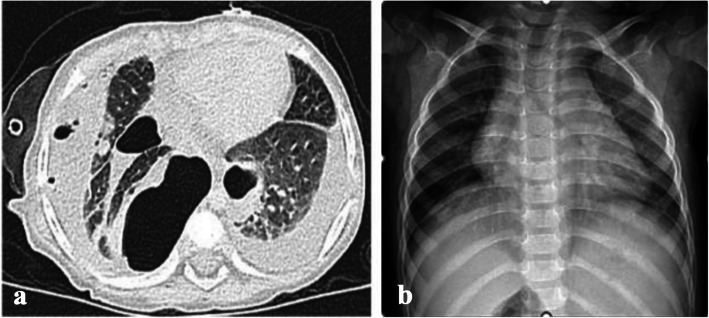
18 months old infant presented with history of high-grade fever, oral thrush and dyspnea for 2 weeks. She presented to the A&E with cyanosis and marked respiratory distress. She had tension pneumothorax on the right side requiring urgent decompression, followed by chest drain insertion. Pleural fluid culture showed growth of MRSA and sputum culture showed growth of Klebsiella pneumoniae. **a **CT chest showed bilateral necrotizing pneumonia. Immunodeficiency, HIV and TB were excluded. She showed good clinical recovery after 3 weeks of IV antibiotics, with regression of inflammatory markers and radiological improvement. **b** The pulmonary lesions resolved completely at the 1 year follow up

**Fig. 10:**
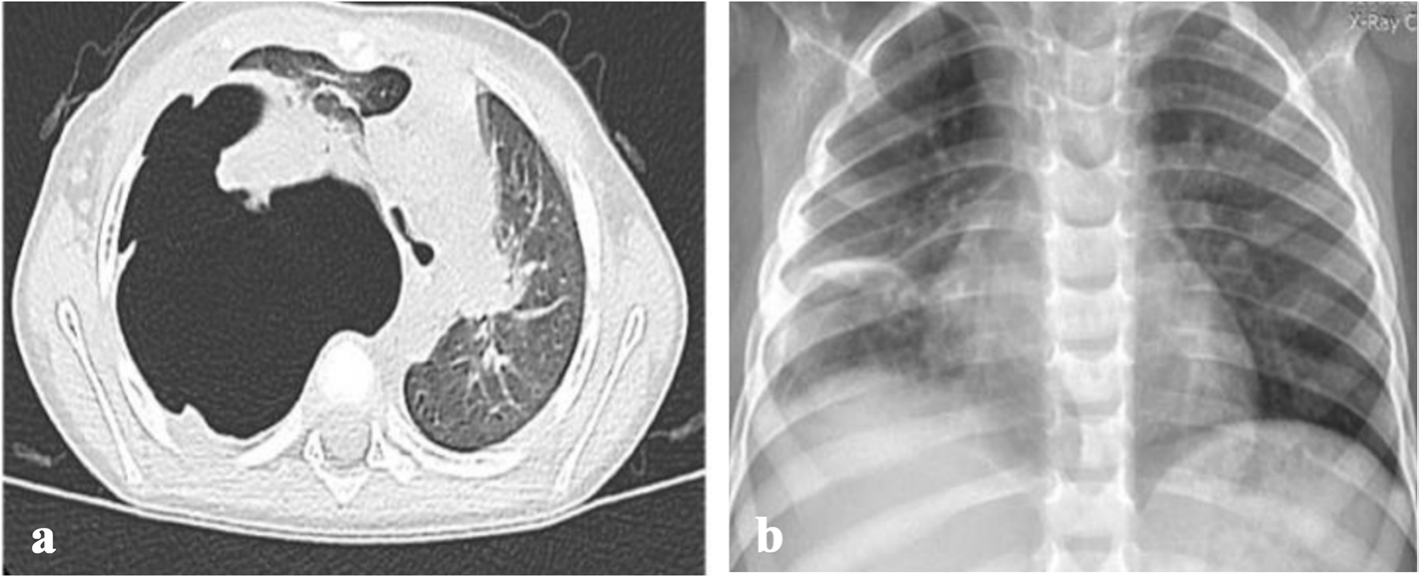
3-year-old child presented with **a**) extensive necrotising pneumonia. Follow up X-ray at 1 year showed marked improvement

**Fig. 11:**
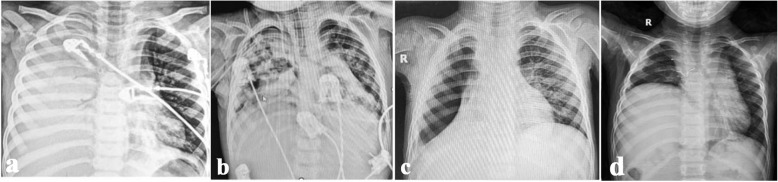
3-year-old child presented with **a**) severe respiratory distress, pancytopenia, rapidly progressive consolidation and highly septated effusion. Respiratory support escalated quickly form high flow nasal cannula to high frequency oscillatory ventilation in 12 h. Two days later, laboratory picture changed to leukocytosis, severe anemia and extreme thrombocytosis. Fibrinolytics were not administered as the patient was hemodynamically unstable. Nasopharyngeal respiratory panel by multiplex PCR was positive for adenovirus, *H influenza and S. pneumoniae.* Pleural fluid and endotracheal aspirate cultures showed no growth of organisms. Blood culture was positive for Staphylococcus Hominis. After 60 days of hospital admission, he had bronchopleural fistula, persistent air leak and lung collapse intractable to drainage and suction (**b**, **c**). He was referred for surgery 3 months after discharge. Decortication and right lower lobectomy were performed. Follow up chest X-ray 2 months after surgery **d**) shows compensatory hyperinflation on the contralateral side

All patients were reviewed in outpatient clinic 2–3 weeks after discharge. Only one patient needed readmission due to progression of PALS on CXR with lung collapse. Long term follow-up, 6–12 months after discharge, was done in patients who had BPF with prolonged hospital admission, and those who had surgery (Figs. 9, 10 and 11).

## Discussion

This retrospective cohort study identified 158 children hospitalized with cCAP over the period of 5 years in the Children’s hospital, Ain Shams University, Cairo- Egypt. Median length of hospital stay (LOS) was 17 days. The median LOS was variable in similar studies, ranging from 9 up to 19.5 days [18, 22–24]. The great majority of children in our study were previously healthy. The clinical characteristics and laboratory data of the study cohort were similar to other published reports [18, 22–24].

More than half of the identified patients had evidence of NP on imaging. Recent epidemiological data demonstrated a rising trend in incidence of cCAP in children, especially NP. This is likely the result of natural evolution of bacterial pathogens and improved sensitivity of imaging modalities [6]. NP is a distinct complication of cCAP and it is crucial to differentiate between NP and pleural infections (CPPE or empyema), as this can impact treatment plans [8, 22].

Children presented with NP had a more complicated hospital course compared to children presented with CAP and empyema or CPPE. NP cohort had a significantly longer LOS, duration of chest drainage, PICU admissions and surgical interventions. NP was associated with CPPE or empyema in most of the children, and this finding was observed in other studies [8, 22]. This potentially explain the high morbidity among this group as they had 2 co-existing CAP complications.

The yeild of pleural, sputum and blood cultures were low among our study cohort (23%, 18% and 17%, respectively), although it is similar to what was reported in similar studies [18, 23–25]. Pleural fluid polymerase Chain Reaction (PCR) improves pathogen identification in pleural fluid up to 95% [25–27], however, it is expensive and was not available for wide use in our hospital.

There is a global lack of regular reporting and analysis of cCAP microbial patterns to fully understand the geographical variations, and data from low- and middle-income countries is particularly scarce [6]. Multiple reports from developed countries demonstared the reduction of IPD after the introduction of PCV. There are also recent reports of increasing incidence of *S pyogens*, *S aureus* cCAP in Europe and USA [28–31]. Despite these reports, *S pneumoniae* remains the lead cause cCAP *worldwide* [16–20].

Review of global patterns of pneumococcal disease, particularly in relation to pneumococcal conjugate vaccine (PCV) use is critical. In high-income countries where PCV13 is routinely administered, the persistence of serotype 3, despite its inclusion in the vaccine, has been implicated in sustained morbidity and vaccine breakthrough cases, as observed in Portugal [32]. Similarly, U.S. data show an initial decline in pneumonia hospitalizations post-PCV13, followed by a modest resurgence, suggesting shifts in disease epidemiology [33]. In contrast, low- and middle-income countries show heterogeneous impacts. For instance, Mongolia reported significant reductions in pneumonia hospitalizations following PCV13 introduction, though non-PCV13 serotype carriage increased [34]. A systematic review across 20 low- and middle-income countries confirmed reductions in vaccine-type carriage, but highlighted wide variability in outcomes [35]. Furthermore, modeling suggests PCV13 could prevent over 697,000 deaths and 131 million cases of pneumococcal disease in low- and middle-income countries by 2030 [36].

These findings highlight the importance of ongoing microbiological surveillance of CAP and cCAP to inform targeted vaccination programs, optimize antimicrobial use, and guide public health interventions aimed at reducing the global burden of pneumococcal disease.

Community acquired MRSA was found to be the most common causative organism among this study cohort, followed by *S pneumonaie*, inspite of the fact that pnuemococcal vaccine is not routinely used in Egypt. *S aureus* strains expressing Panton- Valentine leukocidin and alpha-hemolysin toxins were reported to cause severe tissue damage and NP [37, 38]. Egypt has one of the highest *MRSA* prevalance in Africa and reported in recent studies to be as high as 63% [39, 40].

Other organisms isolated from the cultures included Staphylococcus spp. (aureus and non-aureus), Klebsiella Pneumoniae, Acinetobacter, Pseudomonas aeruginosa, Enterococcus and other Streptococcus spp. These organisms were reported in similar studies [6]. We also report unusual organisms, rarely reported in the literature to be associated with childhood cCAP, such as Ochrobactrum spp, Burkholderia cepacia, Candida Albicans, Escherichia coli, Morganella morganii and Sphingomonas Paucimobilis.

The present study reports 5 patients who had COVID-19 associated cCAP. It was unclear whether the cause of empyema or NP is COVID-19 infection or secondary bacterial pnemonia. Similar presentation has been reported [41–43]. As negative cultures doesn’t necessarily exclude bacterial aetiology, all 5 patients received antibiotic therapy with good outcome, except the 3 month old infant who died of complicated post COVID-19 MIS-C and empyema.

Additionally, Post COVID rise in RSV infections and other respiratory viruses was noted since the ease of the lockdown in late 2020 [44]. RSV and influenza viruses are direct risk factors for CAP and its complications [45]. A national survey among Egyptian children, 2 years after the pandemic, showed that influenza and RSV are the most common organisms isolated from nasopharyngeal swabs from 530 children presented with acute respiratory tract infections (25% and 21% respectively) [46]. Among mortalities reported in the present study was a 2-mo old patient who had RSV and MRSA coinfection. Unfortunately, a detailed correlation between viral infection & cCAP could not be performed in this study as multiplex PCR testing for respiratory viruses’ detection was not available for routine use during the whole study period.

There is a global interest in conducting more research to understand role of viral infections in development of complicated pneumonia. Our study showed a significant reduction in cCAP admissions on the year of the lockdown with gradual resurgence in the cases afterwards, and this finding was also reported globally [47]. Public health measures like lockdowns and masking suppressed common pathogens such as RSV and influenza, leading to reduced natural exposure and a concept known as"immunity debt”, a gap in population immunity, especially in young children [48–50]. As restrictions lifted, delayed and intensified outbreaks of these viruses were reported, often off-season and affecting older age groups [48–50]. These factors likely increased pediatric vulnerability to severe infections and complications like cCAP.

Combined senstivity results of the isolated organisms show an alarimg high resistance for antibiotics commonly used in the management of CAP and it’s complications. Gram positive organisms resistance to ceftriaxone and Amoxicillin/Clavulanic acid was high (73% and 74% respectively). As antibitioc choices in cCAP guidelines largely stem from data extrpolated from studies in USA and Europe, establishing an Egyptian network for microbial and antibiotic senstivity reporting is essential to guide antimicrobial treatement.

Great majority of the study cohort receieved antibiotics before admission (85%). On a national level, a more strict guidance regarding use of antibiotics in primary care settings is needed, as this poses a great risk for circulating antimicrobial resistance among the community. There is no set criteria that defines antibitoic failure in cCAP, and this needs to be adressed in future studies. Nevertheless, fever alone shouldn’t be a criteria for antibiotic change, as prolonged fever could be due to pyrogenic products of inflammation and tissue destruction [22].

Existing cCAP guidelines in children are outdated and there are significant variations in practice across the world with regard to antimicrobial therapy, use of pleural fibrinolytics, VATS and surgery [6].

A common practice in our hospital to perform an ultrasound guided thoracocentesis or pig- tail insertion if the patient presented with effusion, and to insert surgical chest drain if the patient presented with pneumothorax or pyo-pneumothorax. Recent studies showed the high efficacy and safety of pigtails in drainage of pneumothorax [51]. More studies are needed to assess whether pigtails are effective in drainage of pyo-pneumothorax especially in the setting of severe NP and associated effusion.

The high incidence of BPF and PALS in children with NP could be attributed to natural progression of the disease or possibly iatrogenic due to chest drains causing more pressure necrosis on the friable lung tissue. Study by Sawicki, et al. [22] showed that length of chest tube drainage is a risk factor for development of BPF. Recent guidance form centers experienced in complicated pneumonia management recommended avoiding chest drains in NP with mild to moderate effusion, and to manage conservatively with antibiotics only [6].

Pleural fibrinolytics efficacy in management of septated PE is already established and its part of pleural infection management guidelines. However, we reported 3 patients who developed necrotising pneumonia, BPF and PALS immediately after fibrinolytics. These patients didn’t have any radiological evidence of NP prior to alteplase administration. Similar findings have been reported in a study by Livingston, M.H. et al*.* [52]. This deterioration after fibrinolytics could be part of disease progression to NP and coincided with fibrinolytics administration. An alternative hypothesis that fibrinolytics destabilized ongoing fibrinous inflammation and thrombosis, and that could have accelerated the progression to NP.

Other complications following intrapleural fibrinolytics among our study cohort were similar to other centers, which reported serious bleeding, low hemoglobin level and hemothorax [52, 53]. Caution should be exercised, considering the bleeding risks associated with fibrinolytic agents.

A general recommendation in cCAP is to avoid fibrinolytics in NP to prevent severe hemorrhage from the necrotic lung tissue. We report 3 patients presented with NP and highly septated effusion and they were successfully managed with fibrinolytics. These patients had milder parenchymal disease. Radiological scoring of NP severity is needed in future research studies. NP severity needs to be correlated with the safety of fibrinolytic administration to manage the associated pleural infection, and the potential risk of fibrinolytics on worsening parenchymal disease.

More studies are required to assess the role of adjuvant therapies on the clincial outcomes of cCAP. Most of the study cohort had thrombocytosis at a certain point of their illness (98%). Antiplatelets (Aspirin 3 mg OD) was used as an adjuvant therapy to antibiotics and drainage in seven patients who had extensive NP, BPF and severe thrombocytosis (platelet count > 900 × 109/L). Thrombocytosis is frequently observed in children with cCAP, and currently it is considered mainly as an inflammatory marker of the disease. Recent studies linked neutrophil-platelet activation to vascular occlusions, alveolar capillary destruction and necrotizing pneumonia caused by *MRSA and S Pnemoniae* [54]. Further studies are needed to correlate the severity of thrombocytosis and clinical outcomes of cCAP in children, and whether platelets can be a potential treatment target especially in the patients who develop severe or extreme thrombocytosis. Furthermore, the safety of antiplatelets in clinical context of cCAP in children is also unknown.

Complicated pneumonia pathogenesis involves a cascade of inflammation triggered by infection and NP specifically cannot be prevented despite appropriate antibiotics [8]. This hypothetically justifies the use of corticosteroids in cCAP. Till now, corticosteroids are not part of standard management of cCAP as only few RCTs assessed the value of dexamethasone on cCAP outcomes [55]. This remains a gap in cCAP management that needs to be addressed in future studies.

### Study limitations

This study is subject to several limitations, including its retrospective design, which may introduce selection and information bias. The potential for overlapping clinical diagnoses could confound the classification of cases. Additionally, the limited sample size reduces statistical power and generalizability. Furthermore, the inability to retrieve complete clinical and laboratory data for patients admitted with lung abscess represents an additional limitation, restricting the comprehensiveness of the analysis of the clinical spectrum of cCAP admissions.

## Conclusions

This study shows the burden of complicated pneumonia in children from one of largest teriatry hospitals in Egypt. Predominant causative organisms for complicated pneumonia were *MRSA* and *S pneumonaie*. Additionally, this study shows the rising incidence of necrotising pneumonia, especially after COVID-19 pandemic. Children presented with NP had significant morbidity and longer LOS compared to children presented with CAP and empyema or CPPE.

Managment of the cCAP can be very challangeing, even for an experienced respiratory paediatrician. For all children presented with cCAP, assessment of presence of NP on admission is crucial, as local therapies for management of the associated effusion such as chest drains and fibrinolytics can potentially worsen the parenchymal disease, causing BPF & PALS. All decisions regarding chest drainage and fibrinolyitcs should be made promptly after discussion with paediatric respiratory, surgery and radiology teams. Decisions regarding antibiotic treatemtent should be involving paediatric infectious disease and microbiology teams.

The noted high resistance to common antibiotics used in cCAP management among this study is also concerning. More efforts are needed to improve microbial detection and reporting from all Egyptian centers managing CAP and its complications, to guide antimicrobial therapy and decrease antimicrobial resistance.

## Supplementary Information

Supplementary Material 1.

## Data Availability

No datasets were generated or analysed during the current study.

## Abbreviations

CAP: Community-acquired pneumonia
cCAP: Complicated community acquired pneumonia
CPPE: Complicated parapneumonic effusion
NP: Necrotising pneumonia
S pneumoniae: Streptococcus pneumoniae
S pyogens: Streptococcus pyogens
S aureus: Staphylococcus aureus
IPD: Invasive pneumococcal disease
BPF: Bronchopleural fistula
PALS: Persistent air leaks
PICU: Paediatric intensive care unit
PCV13: Pneumococcal conjugate vaccine 13
MRSA: Methicillin-resistant Staphylococcus aureus
MIS-C: Multi-system inflammatory syndrome in children
LUS: Lung ultrasound
VATS: Video assisted thoracoscopic surgery
LOS: Length of hospital stay
PCR: Polymerase Chain Reaction
