# Supplementary material for: Childhood necrotising pneumonia, empyema and complicated parapneumonic effusion secondary to community acquired pneumonia: report of 158 cases from a tertiary hospital in Egypt
Source: Respir Res. 2025 Jul 2;26:235. doi: 10.1186/s12931-025-03291-w (PMC12224481; doi:10.1186/s12931-025-03291-w)
Supplement: Supplementary file 1 — Supplementary Material 1. [file 12931_2025_3291_MOESM1_ESM.docx]

|  |  | Cultures sent on admission | | | Repeat cultures during admission | | | |
| --- | --- | --- | --- | --- | --- | --- | --- | --- |
|  | BAL | pleural | Blood | Sputum | Pleural | blood | Sputum | Wound Swab |
| Subject 1 |  | Streptococcus pneumoniae | No growth | No growth | No growth | MDR Acinetobacter | MDR Acinetobacter | No growth |
| Subject 2 |  | No growth | No growth | No growth | MDR Acinetobacter | No growth | No growth | No growth |
| Subject 3 | β-Hemolytic streptococci | No growth | No growth | No growth | No growth | No growth | MDR Pseudomonas | No growth |
| Subject 4 |  | No growth | No growth | No growth | No growth | MDR Acinetobacter | No growth | No growth |
| Subject 5 |  | Non-hemolytic Streptococcus | No growth | No growth | No growth | No growth | MDR Klebsiella | No growth |
| Subject 6 |  | MRSA | No growth | No growth | No growth | No growth | MDR Pseudomonas | No growth |
| Subject 7 |  | MRSA | No growth | No growth | No growth | MDR Acinetobacter | MDR Acinetobacter | No growth |
| Subject 8 |  | No growth | No growth | No growth | No growth | Candida | MDR Klebsiella | No growth |

MDR- Multi drug resistant

Repeat cultures were obtained during admission if the patient showed clinical deterioration or had persistently elevated—or rising—inflammatory markers. In eight patients, the repeat cultures grew a different organism compared to the admission cultures. Specifically, repeat cultures identified multidrug-resistant (MDR) Acinetobacter in 50% (4 of 8), MDR Klebsiella in 25% (2 of 8), and MDR Pseudomonas aeruginosa in 25% (2 of 8).

The authors engaged in several discussions regarding this subgroup. The microbiology team advised that the clinical significance of the repeat culture findings could not be definitively established, as long-stay patients may be asymptomatic colonizers. Furthermore, the relationship between these findings and length of stay (LOS) could not be determined due to multiple confounding factors, including varying antibiotic regimens, differing severity of pneumonia, organism virulence, and the presence of comorbidities.

Initially, 16 subjects were reported in this cohort in the original manuscript. However, following recommendations from the microbiology team, 8 patients with positive skin swabs from chest drainage sites were excluded, as the clinical relevance of these findings was uncertain.

Recalculation of LOS after exclusion of these 8 subjects is 17 days.
